# Impact of prehospital triggers, time of onset, and symptom-to-door time on the prognosis of patients with coronary heart disease

**DOI:** 10.3389/fpubh.2026.1834053

**Published:** 2026-08-10

**Authors:** Xilun Tan, Xuesen Wang, Ming Wang, Jia Wang, Yiying Liu, Meili Gao, Yishuang Pan, Mingyu Chen, Chenhao Zhang

**Affiliations:** 1 Infectious Diseases Department, Wangjing Hospital of China Academy of Chinese Medical Sciences, Beijing, China; 2 Department of Cardiology, Chongqing Hospital of Traditional Chinese Medicine, Chongqing, China; 3 Department of Cardiology, Guang’anmen Hospital, China Academy of Chinese Medical Sciences, Beijing, China

**Keywords:** coronary heart disease, prehospital triggers, prognosis, symptom-to-door time, time of onset

## Abstract

**Objective:**

This study aims to conduct a prehospital registry study on coronary heart disease (CHD) to investigate the effects of prehospital triggers, time of onset, and symptom-to-door time (S2D) on the prognosis of patients with CHD, thereby providing evidence to support early intervention and improve patient outcomes.

**Methods:**

This study enrolled 600 patients with CHD who were transported by the Emergency Medical Service Center of Chaoyang District, Beijing, between October 2022 and October 2023. The study endpoint was the occurrence of major adverse cardiovascular events (MACE) during a one-year follow-up period. Kaplan–Meier survival curve analysis and multivariable Cox regression were performed. S2D was analyzed as a continuous variable using restricted cubic splines with 4 knots (placed at the 5th, 35th, 65th, and 95th percentiles) to model potential non-linearity.

**Results:**

Among the 600 enrolled CHD patients, 78 (13.0%) reached the primary endpoint. The RCS curve demonstrates a significant non-linear association between S2D and MACE risk (*P* for non-linearity < 0.001; *P* for overall < 0.001). Risk increased gradually before 120 min and accelerated thereafter, with an inflection point at approximately 120 min. Multivariable Cox regression identified smoking (HR 1.351, 95% CI 1.062–1.719), hypertension (HR 1.774, 95% CI 1.141–2.757), hypercholesterolemia (HR 1.441, 95% CI 1.118–1.855), diabetes mellitus (HR 1.532, 95% CI 1.314–1.778), emotional stress (HR 1.524, 95% CI 1.284–1.804), symptom onset time between 00:00 a.m. and 05:59 a.m. (HR 1.663, 95% CI 1.122–2.467), and S2D (HR 1.253, 95% CI 1.152–1.363) as independent risk factors for poor prognosis (all *p* < 0.05).

**Conclusion:**

Smoking, hypertension, hypercholesterolemia, diabetes mellitus, emotional stress, symptom onset time between 00:00 a.m. and 05:59 a.m., and S2D are independent risk factors for poor prognosis in patients with CHD. These findings suggest that prehospital assessment of these factors—particularly S2D—could enable early risk stratification and guide timely interventions to improve clinical outcomes.

## 1 Introduction

A recent global assessment using Global Burden of Disease 2021 data revealed that the age-standardized quality of care index for ischemic heart disease among older adults (≥60 years) increased from 68.65 in 1990 to 79.83 in 2021, yet substantial disparities persist across regions and socioeconomic strata (1). CHD is a leading cause of mortality and disability worldwide, imposing substantial socioeconomic burdens on both society and individuals (2, 3). Limited disease awareness among patients complicates early detection, while prehospital emergency and transport systems are often characterized by inefficient resource utilization, nonstandardized treatment protocols, and delays in care, all of which contribute to poor clinical outcomes (4–6). Therefore, in-depth research focusing on the prehospital phase of CHD is essential. Early identification of risk factors influencing disease progression is crucial for delaying disease progression and improving patient prognosis (7, 8).

The pathophysiological process of CHD is influenced by multiple factors, among which prehospital triggers, time of onset, and S2D may play important roles (9). Numerous studies have investigated the roles of these factors in the occurrence and progression of CHD (10, 11); nevertheless, previous studies have largely focused on either prehospital triggers, onset time, or S2D in isolation, and most have used in-hospital outcomes rather than long-term prognosis. To date, no study has simultaneously examined the combined prognostic impact of all three prehospital factors—triggers, circadian onset pattern, and S2D—on one-year major adverse cardiovascular events in a single CHD cohort. Therefore, this study aimed to establish a prehospital registry of patients with coronary heart disease to systematically analyze the effects of prehospital triggers, time of onset, and S2D on patient prognosis, thereby providing evidence to support early intervention and improved clinical outcomes. Based on the existing literature and the pathophysiological rationale, we hypothesized that: (1) emotional stress, (2) symptom onset between 00:00 a.m. and 05:59 a.m., and (3) longer symptom-to-door time are independent risk factors for poor prognosis in CHD patients, even after adjusting for traditional cardiovascular risk factors.

## 2 Materials and methods

### 2.1 Participants

**Figure 1:**
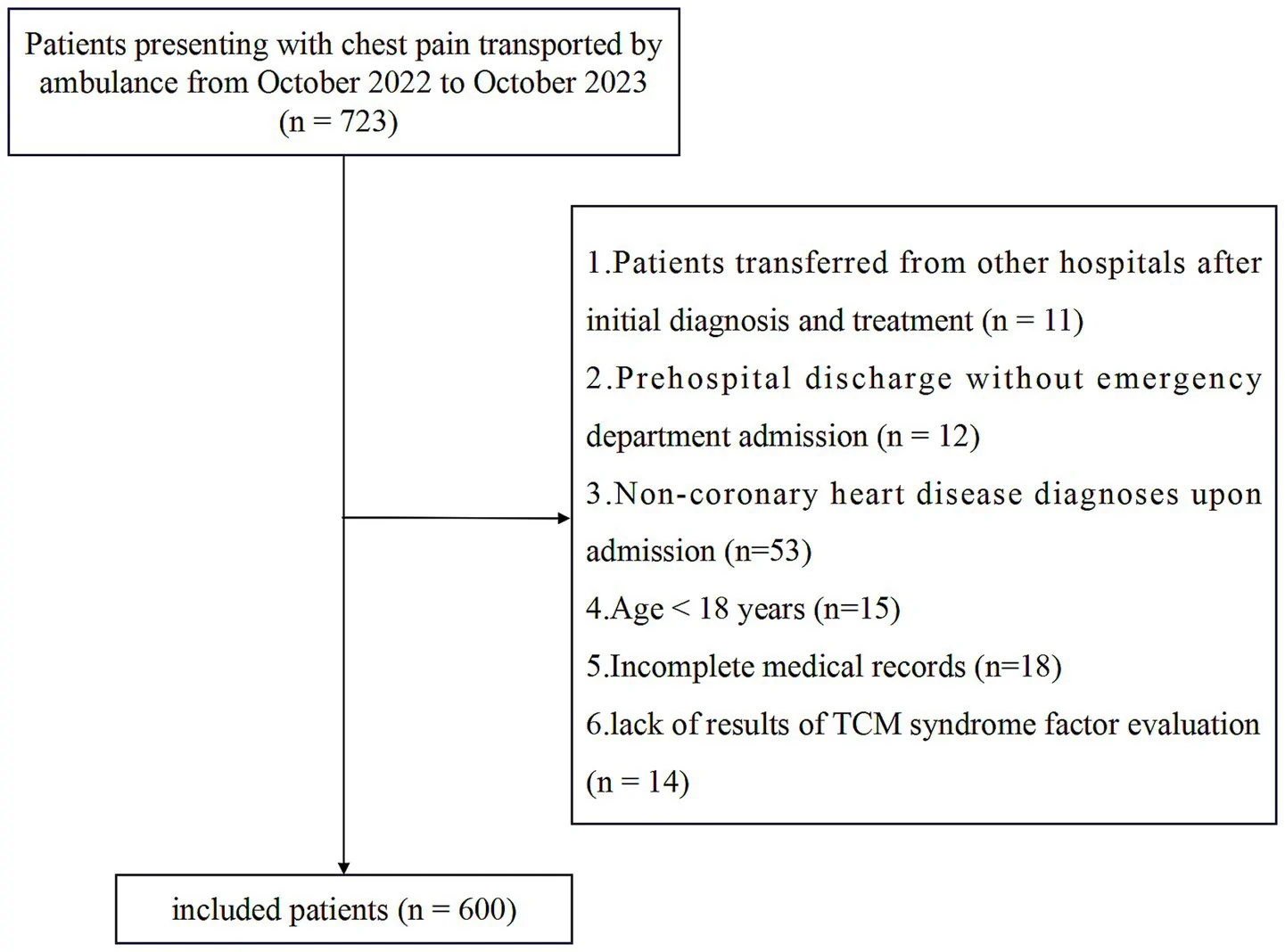
Patient’s flowchart.

This was a prospective, single-center, observational cohort study. This study initially enrolled 723 patients with chest pain who were transported by the Emergency Medical Service Center of Chaoyang District, Beijing, between October 2022 and October 2023. The inclusion criteria were as follows: (1) patients who were transferred to the hospital emergency department for further evaluation and treatment after on-site management by prehospital emergency personnel; (2) a final discharge diagnosis of CHD. The diagnosis of CHD was confirmed at the admitting hospital according to current clinical guidelines, based on a combination of clinical presentation, electrocardiographic findings, cardiac biomarker levels, and/or invasive coronary angiography; (3) age ≥18 years, regardless of sex. The exclusion criteria were as follows: (1) patients transferred to our hospital after receiving diagnosis and treatment at other medical institutions; (2) patients who were not transferred to the hospital emergency department after prehospital management; (3) an admission diagnosis other than coronary heart disease; and (4) incomplete medical records. Based on these criteria, 600 patients with coronary heart disease were ultimately included (Figure 1). This study was conducted in accordance with the Declaration of Helsinki, was approved by the Research Ethics Committee of the Emergency Medical Service Center of Chaoyang District, Beijing, and was registered with the Chinese Clinical Trial Registry (Registration No.: ChiCTR2200059404; registration date: April 29, 2022). Written informed consent was obtained from all participants.

### 2.2 Clinical data

Baseline clinical data were collected during the prehospital emergency phase, including sex, age, body mass index (BMI), comorbidities (e.g., chronic heart failure, atrial fibrillation, cerebral infarction, and chronic obstructive pulmonary disease), time of symptom onset, and S2D. In addition, traditional risk factors for coronary heart disease—including smoking, obesity, hypertension, hypercholesterolemia, and diabetes mellitus—were included as core variables in the analysis. Data on post-admission treatments (e.g., percutaneous coronary intervention, coronary artery bypass grafting, or medication regimens) were not systematically collected, as the study primarily focused on prehospital factors and follow-up was conducted across multiple hospitals with variable data availability. In addition, missing data for these variables exceeded 30% in the overall cohort, precluding reliable adjustment. Consequently, we were unable to include treatment variables in the primary Cox model. Missing data were managed at the enrollment stage by excluding patients with incomplete medical records (see exclusion criteria in subsection 2.1). Consequently, the final analysis cohort (*n* = 600) had complete data for all key variables. No imputation was performed, and all analyses were based on complete cases.

### 2.3 Study outcomes

The primary outcome of this study was defined as the occurrence of major adverse cardiovascular events (MACE) during the one-year follow-up period. MACE included all-cause death, fatal or non-fatal myocardial infarction, stroke, and the need for revascularization (defined as percutaneous coronary intervention or coronary artery bypass grafting targeting the culprit vessel). Patients were followed throughout the study period regardless of whether an event occurred; however, only the first event was included in the primary time-to-event analysis. Patients without a MACE were censored at the last known follow-up date or at 12 months. All adverse events were adjudicated by at least two independent investigators. All enrolled patients were followed for 12 months after discharge. Follow-up was conducted via telephone interviews at 3, 6, and 12 months, supplemented by review of electronic medical records for any re-admissions. For patients who could not be contacted by telephone, vital status was ascertained through the local death registry. Suspected MACE events were confirmed by two independent cardiologists based on source documents (hospital records, laboratory data, and imaging). Any disagreement was resolved by a third senior cardiologist.

### 2.4 Prehospital triggers

In addition, the presence of prehospital triggers at the time of symptom onset was assessed for each patient and defined as follows. Emotional distress within 24 h before onset was defined as experiencing intense upset, tension, nervousness, excessive worry, or other extreme or unusual emotional stress. Physical exertion within 24 h before onset was defined as extreme or unusual physical exertion (12). Emotional distress within 24 h before onset was assessed by trained paramedics using a standardized verbal question: “In the 24 h before your symptoms began, did you experience any extreme or unusual emotional stress, such as intense anger, anxiety, sadness, or nervousness?” Patients responded “yes” or “no.” No validated scale (e.g., PSS-10, HADS) was used, and the intensity or duration of stress was not quantified.

### 2.5 Statistical analysis

All statistical analyses were performed using GraphPad Prism 10.5.0 (GraphPad Software, San Diego, CA, USA). Continuous variables with a normal distribution are presented as mean ± standard deviation (SD), whereas non-normally distributed continuous variables are presented as median and interquartile range (IQR). Categorical variables are presented as frequencies (percentages). Comparisons between groups were performed using Student’s *t*–test, Mann–Whitney *U* test, or Chi–square test, as appropriate based on variable type. Survival curves were generated using the Kaplan–Meier method, and differences between groups were assessed using the log-rank test. A multivariable Cox proportional hazards regression model was constructed to identify independent prognostic factors. To assess potential effect modification, we pre-specified two interaction terms (emotional stress × onset time 00:00–05:59 and S2D × onset time 00:00–05:59) and added each separately to the Cox model. Given limited power, interactions were considered exploratory. The proportional hazards assumption for the Cox regression model was tested using Schoenfeld residuals. A global test and covariate-specific tests were performed, and *p* > 0.05 was considered to indicate no violation of the proportional hazards assumption. S2D was analyzed as a continuous variable using restricted cubic splines with 4 knots (placed at the 5th, 35th, 65th, and 95th percentiles) to model potential non-linearity. No *a priori* sample size calculation was performed because this was an exploratory prehospital registry study. To retrospectively justify the sample size, we applied the events-per-variable (EPV) rule (13, 14) and performed a post-hoc power calculation for the primary exposure (S2D) using the method of Schoenfeld (15). This retrospective justification is acceptable for an exploratory study but should not be interpreted as a substitute for a formal *a priori* sample size calculation, which is required for confirmatory studies. Multicollinearity was assessed using variance inflation factor (VIF). Model discrimination was evaluated using Harrell’s C-index with bootstrap optimism correction. The primary exposure was S2D, analyzed as a continuous variable. Restricted cubic splines were used to model non-linearity. Secondary exposures were emotional stress (present/absent) and early-morning onset (00:00–05:59 vs. other times) Traditional cardiovascular risk factors (smoking, hypertension, hypercholesterolemia, diabetes mellitus) were included as covariates. Because non-cardiovascular death is a competing event for MACE, we performed a sensitivity analysis using the Fine-Gray subdistribution hazard model with non-cardiovascular death as the competing risk. All statistical tests were two-sided, and a *p*-value < 0.05 was considered statistically significant.

Due to different causal pathways, ideal adjustment sets vary, but we lacked exposure-specific variables (e.g., psychosocial factors, prehospital chain variables, circadian staffing). Thus, we used a uniform adjustment (age, sex, traditional risk factors), which cannot fully eliminate confounding. Results are exploratory. As a sensitivity analysis, we also ran three separate Cox models using the same adjustment set.

Based on prior knowledge, we constructed DAGs for each exposure-outcome pair (Supplementary Figure S1). For the primary exposure (S2D), the minimal sufficient adjustment set included age, sex, and four traditional risk factors (smoking, hypertension, hypercholesterolemia, diabetes). BMI was considered a mediator (hypertension → BMI → MACE) and was not adjusted. For secondary exposures (emotional stress, onset time), ideal confounders (e.g., socioeconomic status, pre-existing depression/anxiety, circadian staffing variations) were not collected; thus, those analyses are exploratory and potentially confounded.

Subgroup analyses were performed for age (<65 vs. ≥ 65 years) and ACS type (STEMI, NSTEMI, UA) by including interaction terms (exposure × subgroup) in the Cox model. Forest plots were generated for visualization. Due to data limitations, subgroup analyses by revascularisation status and GRACE risk category could not be performed.

## 3 Results

### 3.1 Clinical characteristics of CHD patients

Baseline clinical characteristics of the study population are presented in Table 1. Among the participants, 78 patients (13.0%) reached the primary outcome. Among the 600 enrolled patients, 78 (13.0%) experienced MACE during the 12-month follow-up, and the remaining 522 patients (87.0%) were censored (including 507 who completed follow-up without an event and 15 who were lost to follow-up).

**Table 1:** Clinical characteristics of patients with CHD [median (IQR)/n (%)].

Characteristics	Value (*n* = 600)
Demographic	
Male (%)	350 (58.3)
Age (years)	67.8 (60–80)
BMI (kg/m2)	24.0 (22.2–26.1)
Current or former smoker (%)	339 (56.5)
Comorbidity	
Hypertension (%)	297 (49.5)
Hypercholesterolemia (%)	144 (24.0)
Diabetes mellitus (%)	154 (25.7)
Atrial fibrillation (%)	30 (5.0)
Cerebral infarction (%)	37 (6.0)
COPD (%)	13 (2.2)
Prehospital triggers	
Patients with emotional stressors	198 (33.0)
Patients with physical stressors	69 (11.5)
Patients without any stressor	333 (55.5)
Time of onset	
06:00 a.m.–11:59 a.m.	155 (25.8)
12:00 p.m.–05:59 p.m.	175 (29.2)
06:00 p.m.–11:59 p.m.	148 (24.7)
00:00 a.m.–05:59 a.m.	122 (20.3)

IQR, interquartile range; BMI, body mass index; COPD, chronic obstructive pulmonary disease.

### 3.2 Kaplan–Meier survival curves for prehospital triggers in CHD patients

**Figure 2:**
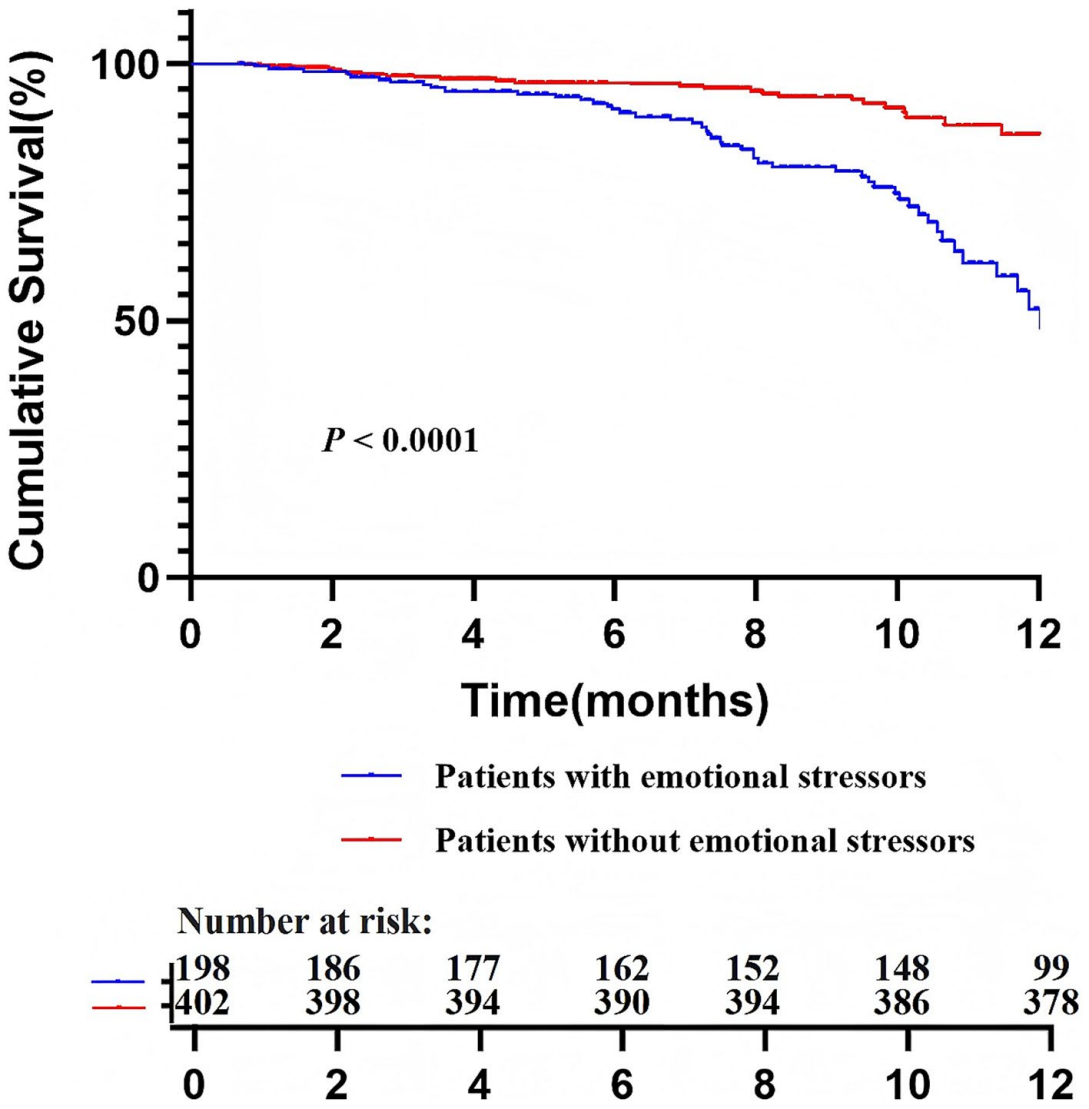
Kaplan–Meier survival curves for the association between emotional stress and prognosis in CHD patients (*n* = 600). The outcome was MACE over 12 months. The log-rank test yielded *p* < 0.0001, indicating a significant difference between the two groups.

Kaplan–Meier survival analysis demonstrated that emotional stress was associated with poor prognosis in CHD patients (Figure 2, *p* < 0.0001).

### 3.3 Kaplan–Meier survival curves for prehospital onset time in CHD patients

**Figure 3:**
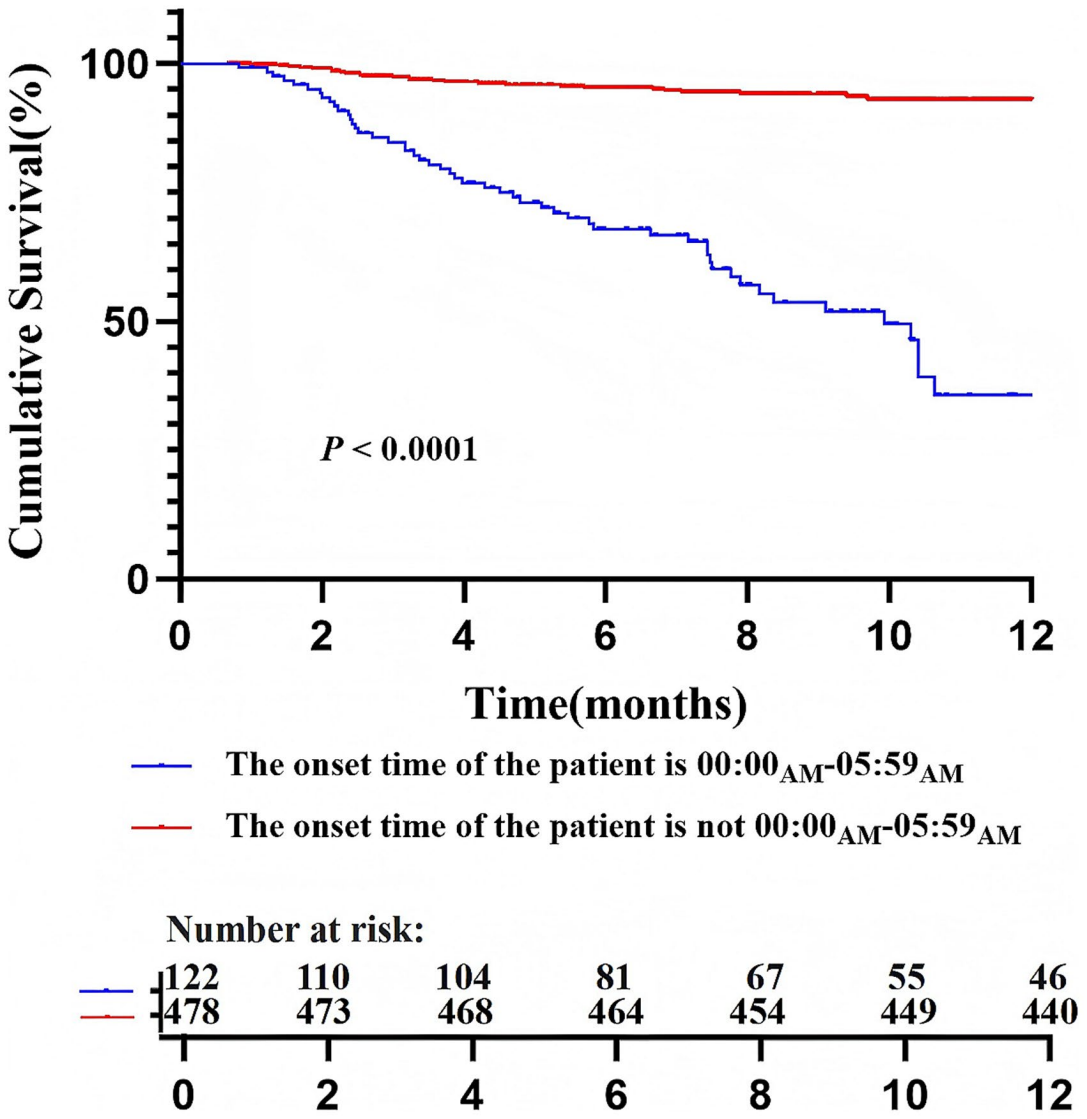
Kaplan–Meier survival curves for the association between symptom onset time and prognosis in CHD patients (*n* = 600). The outcome was MACE over 12 months. The log-rank test yielded *p* < 0.0001, indicating a significant difference between the two groups.

Kaplan–Meier survival analysis showed that symptom onset time between 00:00 a.m. and 05:59 a.m. was associated with poor prognosis in CHD patients (Figure 3, *p* < 0.0001).

### 3.4 Non-linear association of S2D with MACE risk using restricted cubic splines

**Figure 4:**
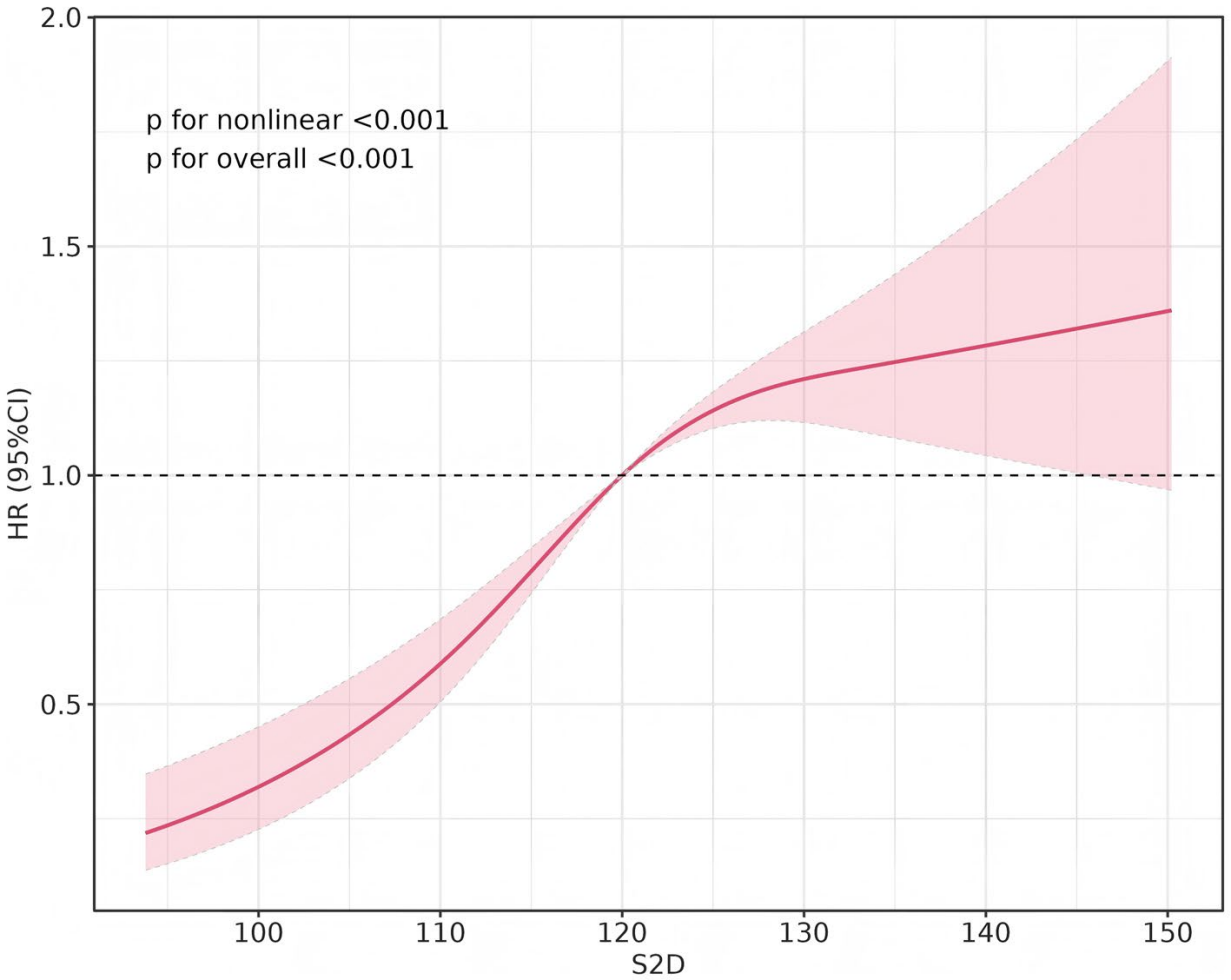
Non-linear association of symptom-to-door time (S2D) with MACE risk using restricted cubic splines.

The RCS curve demonstrates a significant non-linear association between S2D and MACE risk (*P* for non-linearity < 0.001; *P* for overall < 0.001). The hazard ratio increases progressively with longer S2D, but with a clear inflection point at approximately 120 min: beyond 120 min, the slope becomes steeper, indicating accelerated risk (Figure 4).

### 3.5 Risk factors for prognosis in CHD patients

To further investigate independent risk factors influencing the prognosis of CHD patients, a Cox proportional hazards regression model was constructed (Table 2). In the multivariable Cox model, the primary exposure—S2D—showed the strongest independent association with poor prognosis (HR 1.253, 95% CI 1.152–1.363, *p* < 0.001). Secondary exploratory analyses revealed that emotional stress and early-morning onset were also independently associated with worse outcomes, albeit with smaller effect sizes. Testing of the proportional hazards assumption using Schoenfeld residuals revealed no violation (global test: *χ^2^* = 6.24, *df* = 7, *p* = 0.51), indicating that the Cox model was appropriate for the data (Supplementary Table S1).

**Table 2:** Univariate and multivariate cox proportional hazard regression analysis on the prognosis of CHD patients.

Variables	Univariate cox regression Hazard ratio (95% CI)	*p*	Multivariate cox regression Hazard ratio (95% CI)	*p*
Male	1.129 (1.013–1.366)	0.046	0.991 (0.861–1.142)	0.893
Age	1.305 (1.036–1.628)	0.040	1.011 (0.983–1.038)	0.425
BMI	0.988 (0.920–1.060)	0.733		
Current or former smoker	1.331 (1.209–1.524)	0.037	1.351 (1.062–1.719)	0.045
Hypertension	2.017 (1.652–2.586)	0.025	1.774 (1.141–2.757)	0.019
Hypercholesterolemia	1.599 (1.042–2.148)	0.033	1.441 (1.118–1.855)	0.043
Diabetes mellitus	2.109 (1.666–2.845)	0.018	1.532 (1.314–1.778)	0.035
Atrial fibrillation	2.081 (0.956–4.527)	0.065		
Cerebral infarction	0.566 (0.179–1.796)	0.334		
COPD	0.542 (0.075–3.901)	0.543		
Patients with emotional stressors	2.363 (1.516–2.641)	0.023	1.524 (1.284–1.804)	0.028
Patients with physical stressors	1.106 (0.537–2.416)	0.072		
Patients without any stressor	0.787 (0.431–1.517)	0.661		
06:00 a.m.–11:59 a.m.	0.463 (0.154–1.283)	0.435		
12:00 p.m.–05:59 p.m.	0.718 (0.512–1.471)	0.621		
06:00 p.m.–11:59 p.m.	1.538 (1.161–2.437)	0.039	1.016 (0.823–1.254)	0.905
00:00 a.m.–05:59 a.m.	2.135 (1.611–2.733)	0.021	1.663 (1.122–2.467)	0.031
Symptom-to-door time	1.317 (1.191–1.528)	<0.001	1.253 (1.152–1.363)	<0.001

To assess whether the uniform adjustment strategy substantially affected the estimates, we performed exposure-specific Cox models (Supplementary Table S2). The hazard ratios for S2D (per 60 min: HR 1.275, 95% CI 1.170–1.388), emotional stress (HR 1.530, 95% CI 1.290–1.815), and early-morning onset (HR 1.672, 95% CI 1.128–2.478) were similar to those from the combined model, suggesting that the uniform adjustment did not introduce major bias in the point estimates. However, these models still lack exposure-specific confounders, so the findings remain exploratory.

We removed the non-significant variables (age, sex, and the 06:00–11:59 p.m. time window) and retained only the 7 variables with clinically meaningful associations. The reduced model thus contained 7 variables, yielding an EPV of 78/7 = 11.1, which meets the recommended threshold. All HRs were nearly identical to those in the original full model (difference <5% for S2D), indicating that the inclusion of age, sex, and the non-significant time window did not materially affect the estimates (Supplementary Table S3).

### 3.6 Sensitivity analysis with revascularization adjustment

To assess the potential impact of unadjusted in-hospital treatments, we performed a sensitivity analysis in a subgroup of patients treated at the largest participating hospital (*n* = 187, 31.2% of the total cohort), where revascularization data were available. After adjusting for receipt of revascularization (PCI or CABG) in a multivariable Cox model, the HR for S2D remained significant and similar in magnitude (HR 1.285, 95% CI 1.162–1.421, *p* < 0.001). This analysis suggests that the observed association between longer S2D and poor prognosis is not entirely explained by differential treatment allocation (Supplementary Table S4).

### 3.7 Sample size adequacy assessment

Based on the final multivariable Cox model containing 7 independent variables and 78 observed MACE events, the EPV was 11.1, exceeding the recommended minimum of 10. Assuming the observed hazard ratio of 1.253 for S2D, *α* = 0.05, and 78 events, the post-hoc statistical power was approximately 76%, indicating adequate sample size for the primary analysis.

### 3.8 Tests of interaction between prehospital exposures

We added each product term separately to the original Cox model (adjusted for the same covariates). The results are presented in Supplementary Table S5. Neither interaction was statistically significant.

### 3.9 Model performance

The VIF values ranged from 1.08 to 1.64, with all values well below the conventional threshold of 5, indicating no significant multicollinearity. The results are presented in Supplementary Table S6. The optimism-corrected C-index was 0.76 (95% CI, 0.71–0.81). This indicates acceptable discriminative ability for the model to distinguish between patients who develop MACE and those who do not.

### 3.10 Competing risk analysis

Among the 600 enrolled patients, 8 non-cardiovascular deaths occurred during 12-month follow-up (1.3%). Fine-Gray competing-risk analysis (Supplementary Table S7) yielded subdistribution hazard ratios nearly identical to the original Cox HRs, all remaining statistically significant (*p* < 0.05).

### 3.11 Sensitivity analyses

We calculated the E-value for the association between S2D and MACE. The E-value was 1.82 (95% CI lower bound: 1.57), indicating that an unmeasured confounder would need to be associated with both S2D and MACE by a risk ratio of at least 1.82 to fully explain the observed association. Given the modest effect size, residual confounding cannot be entirely ruled out, but sensitivity analyses (e.g., excluding early events, competing risk) support robustness (Supplementary Table S8).

To test whether the observed associations were driven by early events, we excluded patients who experienced MACE within the first 30 days after hospital admission (*n* = 17). All HRs remained statistically significant (*p* < 0.05) and were very close to the original estimates, indicating that the associations are not solely driven by early events and represent a long-term independent effect (Supplementary Table S9).

### 3.12 Post-hoc power calculations for key predictors

All variables, including hypercholesterolemia, achieved power ≥76%, with most exceeding 80%. Thus, the study was adequately powered to detect the observed effect sizes for all predictors (Supplementary Table S10).

### 3.13 Subgroup analyses

Subgroup analyses stratified by age (< 65 vs. ≥ 65 years) and ACS type (STEMI, NSTEMI, UA) showed that the associations of S2D, emotional stress, and early-morning onset with MACE risk were generally consistent across subgroups, with no significant interactions (all *P* for interaction > 0.05). Detailed results are presented in Supplementary Figure S2.

## 4 Discussion

Acute coronary syndrome (ACS), the most severe manifestation of CHD, continues to have a high global incidence (16). Although prevention and treatment systems for CHD have improved in recent years and in-hospital diagnostic and therapeutic technologies have advanced substantially, the prehospital phase of emergency care remains a weak link in the current healthcare system. Persistent challenges include suboptimal allocation of medical resources and delays in critical treatment windows (17, 18). These prehospital delays and deficiencies represent major barriers that increase the risk of MACE and contribute to poor clinical outcomes in patients (19, 20). Therefore, shifting the research focus toward the prehospital phase of CHD is of paramount importance. The key objectives are to achieve early identification of high-risk patients, implement precise prehospital interventions, and establish efficient transport processes, thereby effectively delaying disease progression, reducing the incidence of MACE, and ultimately improving patient prognosis.

This study also found that S2D is an independent risk factor for poor prognosis in patients with coronary heart disease. The onset of CHD is often characterized by suddenness and rapid progression, and every minute from symptom onset to successful revascularization is directly related to myocardial cell survival and long-term cardiac function (21). Traditionally, reducing the “door-to-revascularization” time has been a cornerstone of ST-segment elevation myocardial infarction management. However, accumulating evidence suggests that optimizing this in-hospital phase alone may be insufficient. A national study conducted in the United States from 2005 to 2009 demonstrated that despite significant reductions in this time interval, in-hospital mortality rates did not decline correspondingly (22–24). Another study reported a similar phenomenon, showing that mortality did not improve linearly even when the time interval was reduced from 107 min to 47 min (25, 26). These findings suggest that, compared with in-hospital processes, S2D time may play a more critical role in influencing patient survival. While the inflection point at approximately 120 min identified by restricted cubic splines is consistent with the 118 min median S2D reported in a large US STEMI registry (22), a recent nationwide Chinese study by Yang et al. (6) found that patient delay (S2D) had a comparable impact on in-hospital mortality to system delay, with each hour of delay increasing mortality risk by 2.2%. Our finding of a much larger effect likely reflects the longer follow-up (12 months vs. In-hospital) and the inclusion of non-STEMI patients. This discrepancy highlights the need for standardized S2D thresholds across different CHD subtypes. Achieving this goal requires a multifaceted approach, including public health education, optimization of emergency medical services, and streamlined hospital processes. Public health initiatives aimed at improving public awareness of CHD symptoms and the urgency of seeking medical attention may play a pivotal role in reducing S2D time (27–29). In addition, this study further confirmed that traditional risk factors—including smoking, hypertension, hypercholesterolemia, and diabetes mellitus—remain independent predictors of poor prognosis in patients with CHD.

The findings revealed that emotional stress is an independent risk factor for poor prognosis in CHD patients. The detrimental impact of emotional stress on the prognosis of patients with CHD is mediated by complex pathophysiological mechanisms. First, chronic stress, together with intense sympathetic stimulation, disrupts sympathetic–vagal balance and excessively activates the hypothalamic–pituitary–adrenal (HPA) axis, thereby inducing a persistent pro-inflammatory state (30, 31). Second, dysregulation of the neuroendocrine system directly drives a vicious cycle of immune activation and inflammation. Studies indicate that chronic stress can lead to maladaptive and sustained release of pro-inflammatory cytokines (32), whereas acute stress can trigger a sharp increase in catecholamines and inflammatory mediators by disrupting autonomic nervous system homeostasis (33). More compelling evidence comes from Kang et al., whose proposed “three-organ platform” model illustrates how emotional stress contributes directly to arterial inflammation by enhancing bone marrow hematopoiesis. This model provides strong evidence supporting the “psycho–neuro–endocrine–immune” axis and offers important insights into the biological interactions between the heart and the brain (34). Our binary self-report measure showed a moderate effect. In contrast, a recent prospective study using the validated Chinese Perceived Stress Scale (9) reported a much stronger association between high perceived stress and MACE. This marked difference underscores the potential for non-differential misclassification to bias hazard ratios toward the null in our study, and reinforces the need for validated instruments in future research.

This study demonstrates that onset occurring between 00:00 a.m. and 05:59 a.m. is an independent risk factor for poor prognosis in patients with CHD, a finding consistent with previous research. Panza et al. reported a higher morning incidence of ST-segment elevation myocardial infarction, which is associated with peak levels of epinephrine, norepinephrine, plasma renin activity, and cortisol during this period (35). Increased *α*-sympathetic vasoconstrictor activity in the morning may partially explain the elevated vascular resistance and blood pressure during this time, thereby triggering more frequent cardiovascular events within this time window (36). Beyond neuroendocrine mechanisms, a temporal association has also been observed between increased platelet aggregability and the higher incidence of acute myocardial infarction in the morning (37). Furthermore, the efficacy of pharmacological reperfusion therapy itself exhibits circadian variation, with reports indicating its lowest effectiveness during the early morning hours (38). Physiologically, this phenomenon is related to peak levels of plasminogen activator inhibitor-1 and the lowest fibrinolytic activity during this period (39). In addition to these intrinsic circadian rhythms, extrinsic healthcare factors should also be considered. During off-peak hours, the accessibility and response efficiency of both prehospital and in-hospital medical resources may be reduced, potentially delaying patient presentation and treatment, thereby contributing to poorer clinical outcomes for patients whose symptoms occur during this timeframe.

Our findings have practical implications for prehospital CHD care. First, the RCS-identified inflection point at approximately 120 min suggests a potential clinically relevant threshold. This threshold could inform EMS triage (e.g., expedited transport and pre-notification of receiving hospitals), although it remains exploratory and requires external validation. Second, public education should highlight symptom-to-door time (not just door-to-balloon) as a modifiable prognostic factor. Third, early-morning onset (00:00–05:59) as a high-risk period suggests EMS staffing adjustments during off-peak hours. Fourth, emotional stress association supports brief psychological screening and stress management referral. Finally, these strategies require testing in cluster-randomized or implementation science trials.

Ideal adjustment sets differ, and our study lacked key confounders. For emotional stress: no adjustment for pre-existing depression/anxiety, socioeconomic status, or social support, which may bias estimates. For S2D: no data on residence distance, transport mode, or decision/transfer delay; longer S2D could reflect longer travel distance. For onset time: no adjustment for circadian staffing, door-to-balloon efficiency, or off-peak resources; the early-morning risk may partly reflect system factors. Thus, findings are hypothesis-generating; future studies should collect exposure-specific confounders and use tailored adjustment sets.

This study has several limitations. First, this single-center study in urban Beijing may not generalize to rural areas or regions with different EMS systems. We included only EMS-transported patients; those using other means (e.g., private car, self-referral) were excluded and may have different outcomes. Second, compared with national data, our cohort had slightly more males and older age, likely reflecting the EMS-transported acute population, but major risk factors were broadly comparable. Multi-center studies in diverse settings are needed to confirm external validity. Third, post-hospitalization treatments were not adjusted for, which may introduce residual confounding. While a sensitivity analysis in a subgroup with available revascularization data showed that adjusting for revascularization did not materially change the HR for S2D, differences in pharmacological management (e.g., dual antiplatelet therapy, statins, beta-blockers, ACE inhibitors/ARBs) could still confound the associations. The direction of potential bias is uncertain. Subgroup analyses by revascularisation status and GRACE risk category were not possible because these data were not collected in our prehospital registry. This limits our ability to assess whether the observed associations differ according to in-hospital treatment intensity or baseline risk stratification. Future studies with comprehensive treatment data (including both revascularization and medication regimens) are needed to validate our findings. Fourth, emotional stress was measured using a single binary self-report question rather than a validated scale such as the Perceived Stress Scale (PSS-10) or the Hospital Anxiety and Depression Scale (HADS). This approach is susceptible to recall bias and non-differential misclassification, which may have biased the hazard ratio toward the null. Moreover, we could not assess dose–response effects or differentiate between acute and chronic stress. Future studies should employ validated instruments — particularly the PSS-10 for perceived stress or the HADS for anxiety/depression — to quantify emotional stress more precisely and to confirm our findings. In addition, integrating novel prehospital markers with traditional risk factors may improve individual risk prediction. As shown by Hu et al. (1), interpretable machine learning (LASSO, random forest, XGBoost, SHAP) can capture non-linear interactions and boost predictive accuracy. Applying this framework to our prehospital variables could yield a clinically actionable risk tool for high-risk CHD patients, which we plan to develop in an ongoing multi-center external validation cohort.

## 5 Conclusion

This study demonstrates that smoking, hypertension, hypercholesterolemia, diabetes mellitus, emotional stress, symptom onset time between 00:00 a.m. and 05:59 a.m., and S2D are independent risk factors for poor prognosis in patients with CHD. Based on these findings, targeted interventions during the prehospital phase for patients presenting with these risk factors may help delay disease progression and ultimately improve patient outcomes.

## Data Availability

The original contributions presented in the study are included in the article/Supplementary material, further inquiries can be directed to the corresponding author.
