# Supplementary material for: Impact of prehospital triggers, time of onset, and symptom-to-door time on the prognosis of patients with coronary heart disease
Source: Front Public Health. 2026 Aug 10;14:1834053. doi: 10.3389/fpubh.2026.1834053 (PMC13500482; doi:10.3389/fpubh.2026.1834053)
Supplement: Supplementary file 1 [file Data_Sheet_1.docx]

Supplementary Table S1. Testing of the proportional hazards assumption using Schoenfeld residuals for the multivariable Cox regression model

| Variable | *χ²* | *df* | *P* value |
| --- | --- | --- | --- |
| Smoking | 0.82 | 1 | 0.36 |
| Hypertension | 1.15 | 1 | 0.28 |
| Hypercholesterolemia | 0.31 | 1 | 0.58 |
| Diabetes mellitus | 0.96 | 1 | 0.33 |
| Emotional stress | 1.88 | 1 | 0.17 |
| Onset time 00:00–05:59_AM_ | 0.44 | 1 | 0.51 |
| S2D | 0.68 | 1 | 0.41 |
| Global | 6.24 | 7 | 0.51 |

A *P* value > 0.05 indicates no violation of the proportional hazards assumption. The global test assesses the overall model fit.

Supplementary Table S2. Exposure‑specific Cox regression models for each prehospital factor

| Exposure | Model adjustment | HR | 95% CI | *P* value |
| --- | --- | --- | --- | --- |
| S2D | Age, sex, smoking, hypertension, hypercholesterolemia, diabetes | 1.275 | 1.170–1.388 | <0.001 |
| Emotional stress | Age, sex, smoking, hypertension, hypercholesterolemia, diabetes | 1.530 | 1.290–1.815 | 0.027 |
| Onset time 00:00–05:59_AM_ | Age, sex, smoking, hypertension, hypercholesterolemia, diabetes | 1.672 | 1.128–2.478 | 0.030 |

These models use the same covariate set but are fitted separately for each exposure to avoid mutual adjustment.

Supplementary Table S3. Reduced Cox model (7 variables, EPV = 11.1)

| Variable | HR | 95% CI | *P* value |
| --- | --- | --- | --- |
| S2D | 1.266 | 1.155–1.375 | <0.001 |
| Emotional stress | 1.520 | 1.280–1.805 | 0.028 |
| Onset time 00:00–05:59_AM_ | 1.660 | 1.120–2.460 | 0.031 |
| Smoking | 1.351 | 1.062–1.719 | 0.045 |
| Hypertension | 1.774 | 1.141–2.757 | 0.019 |
| Hypercholesterolemia | 1.441 | 1.118–1.855 | 0.043 |
| Diabetes | 1.532 | 1.314–1.778 | 0.035 |

Supplementary Table S4. Sensitivity analysis for the association between S2D and prognosis in a subgroup of patients with available revascularization data (n=187).

| Model | HR for S2D | 95% CI | *P* value |
| --- | --- | --- | --- |
| Unadjusted for revascularization | 1.310 | 1.185–1.448 | <0.001 |
| Adjusted for revascularization | 1.285 | 1.162–1.421 | <0.001 |

S2D, symptom-to-door time; HR, hazard ratio; CI, confidence interval.

Supplementary Table S5. Tests of interaction between prehospital exposures

| Interaction term | Subgroup size (with both exposures) | HR for interaction | 95% CI | *P* value |
| --- | --- | --- | --- | --- |
| Emotional stress × Onset time 00:00–05:59_AM_ | 31 | 1.210 | 0.680–2.150 | 0.52 |
| S2D × Onset time 00:00–05:59_AM_ | 43 | 1.080 | 0.852–1.369 | 0.55 |

Model adjusted for age, sex, smoking, hypertension, hypercholesterolemia, diabetes, and the main effects of the two interacting variables. Interaction terms were added separately to avoid overfitting.

Supplementary Table S6. Variance inflation factor (VIF) for variables in the final Cox model

| Variable | VIF |
| --- | --- |
| Age | 1.12 |
| Sex | 1.08 |
| Smoking | 1.21 |
| Hypertension | 1.64 |
| Hypercholesterolemia | 1.15 |
| Diabetes | 1.33 |
| Emotional stress | 1.18 |
| Onset time 00:00–05:59_AM_ | 1.26 |
| Onset time 06:00–11:59_PM_ | 1.31 |
| S2D | 1.42 |

Supplementary Table S7. Fine-Gray competing-risk model (non-cardiovascular death as competing event)

| Variable | SHR | 95% CI | P value |
| --- | --- | --- | --- |
| S2D | 1.248 | 1.145–1.358 | <0.001 |
| Emotional stress | 1.514 | 1.276–1.795 | 0.035 |
| Onset time 00:00–05:59_AM_ | 1.654 | 1.115–2.453 | 0.031 |
| Smoking | 1.345 | 1.058–1.709 | 0.046 |
| Hypertension | 1.769 | 1.137–2.752 | 0.019 |
| Hypercholesterolemia | 1.438 | 1.115–1.851 | 0.043 |
| Diabetes | 1.526 | 1.308–1.772 | 0.035 |
| Age | 1.011 | 0.983–1.038 | 0.613 |
| Sex (male) | 0.991 | 0.861–1.142 | 0.892 |
| Onset time 06:00–11:59_PM_ | 1.016 | 0.823–1.254 | 0.875 |

The Fine-Gray model uses the same covariate set as the primary Cox model. Non‑cardiovascular death (n=8) was treated as the competing event. SHR, subdistribution hazard ratio.

Supplementary Table S8. E-value for unmeasured confounding (S2D → MACE)

| Parameter | Value |
| --- | --- |
| Observed HR | 1.253 |
| Lower 95% CI bound | 1.152 |
| E‑value (point estimate) | 1.82 |
| E‑value (CI lower bound) | 1.57 |

Supplementary Table S9. Cox model after excluding patients with MACE within the first 30 days (n=583, events=61)

| Variable | HR | 95% CI | P value |
| --- | --- | --- | --- |
| S2D | 1.235 | 1.128–1.352 | <0.001 |
| Emotional stress | 1.495 | 1.252–1.775 | 0.034 |
| Onset time 00:00–05:59_AM_ | 1.632 | 1.082–2.432 | 0.026 |
| Smoking | 1.317 | 1.025–1.679 | 0.042 |
| Hypertension | 1.725 | 1.105–2.693 | 0.018 |
| Hypercholesterolemia | 1.408 | 1.082–1.816 | 0.041 |
| Diabetes | 1.483 | 1.272–1.730 | 0.033 |
| Age | 1.016 | 0.983–1.041 | 0.58 |
| Sex (male) | 0.984 | 0.842–1.150 | 0.79 |
| Onset time 06:00–11:59_PM_ | 1.022 | 0.822–1.271 | 0.81 |

Model includes the same covariates as the primary analysis. HRs and CIs are re-estimated based on the reduced sample (excluding 17 patients with MACE within 30 days).

Supplementary Table S10. Post-hoc power calculations for key predictors

| Variable | Expected events | Observed HR | Power (α=0.05) |
| --- | --- | --- | --- |
| S2D | N/A | 1.253 | 76% |
| Smoking | 44 | 1.351 | >95% |
| Hypertension | 39 | 1.774 | >95% |
| Hypercholesterolemia | 19 | 1.441 | 81% |
| Diabetes | 20 | 1.532 | 85% |
| Emotional stress | 26 | 1.524 | 88% |
| Onset time 00:00–05:59_AM_ | 16 | 1.663 | 78% |

Supplementary Figure S1. Directed acyclic graphs (DAGs) for the associations of (A) S2D, (B) emotional stress, and (C) onset time with MACE in CHD patients.

Supplementary Figure S2. Forest plots of subgroup analyses for the association of prehospital exposures with MACE risk. (A) Symptom-to-door time (S2D); (B) Emotional stress; (C) Onset time 00:00–05:59
